# Supplementary material for: Distinct SARS-CoV-2 antibody reactivity patterns in coronavirus convalescent plasma revealed by a coronavirus antigen microarray
Source: Sci Rep. 2021 Apr 6;11:7554. doi: 10.1038/s41598-021-87137-7 (PMC8024395; doi:10.1038/s41598-021-87137-7)
Supplement: Supplementary file 1 — Supplementary Information 1. [file 41598_2021_87137_MOESM1_ESM.pdf]

Distinct SARS-CoV-2 Antibody Reactivity Patterns in Coronavirus Convalescent Plasma Revealed by a Coronavirus Antigen Microarray

Rafael Assis[1], Aarti Jain[1], Rie Nakajima[1], Algis Jasinskas [1], Saahir Khan[2], Huw Davies[1], Laurence Corash[3], Larry J. Dumont[4,5,6], Kathleen Kelly[5], Graham Simmons[7], Mars Stone[7], Clara Di Germanio[7], Michael Busch[7,8], Philip L. Felgner * [1].

Affiliations

1. Vaccine Research and Development Center, Department of Physiology and Biophysics, School of Medicine, University of California, Irvine, CA
2. Division of Infectious Diseases, Department of Medicine, Keck School of Medicine, University of Southern California, Los Angeles, CA
3. Cerus Corporation, Concord, CA
4. University of Colorado School of Medicine, Aurora, CO
5. Vitalant Research Institute, Denver, CO
6. Geisel School of Medicine at Dartmouth, Lebanon, NH
7. Vitalant Research Institute, San Francisco, CA
8. University of California, San Francisco, San Francisco, CA

Corresponding author: pfelgner@hs.uci.edu

Supplementary Table 1

COVAM antigens in order displayed on the figures. All antigens were supplied by Sino Biological Inc.

Antigen ID	Virus	Strain	Protein	Catalogue No
SARS.CoV.2.NP	SARS-CoV-2	SARS-CoV-2	NP	40588-V08B
SARS.CoV.2.Pl.pro	SARS-CoV-2	SARS-CoV-2	Papain-like protease	40593-V07E
SARS.CoV.2.S1	SARS-CoV-2	SARS-CoV-2	S1 subunit	40591-V08B1
SARS.CoV.2.S1.HisTag	SARS-CoV-2	SARS-CoV-2	S1, (His Tag)	40591-V08H
SARS.CoV.2.S1.mFcTag	SARS-CoV-2	SARS-CoV-2	S1, (mFc Tag)	40591-V05H1
SARS.CoV.2.S1.RBD	SARS-CoV-2	SARS-CoV-2	S1-RBD	40592-V05H
SARS.CoV.2.S1+S2	SARS-CoV-2	SARS-CoV-2	S1+S2	40589-V08B1
SARS.CoV.2.S2	SARS-CoV-2	SARS-CoV-2	S2	40590-V08B
SARS.CoV.2.Spike.RBD.Bac	SARS-CoV-2	SARS-CoV-2	RBD	40592-V08B
SARS.CoV.2.Spike.RBD.His.HEK	SARS-CoV-2	SARS-CoV-2	RBD	40592-V08H
SARS.CoV.2.Spike.RBD.rFc	SARS-CoV-2	SARS-CoV-2	RBD	40592-V31H
SARS.CoV_NP	SARS-CoV-2	SARS	NP	40143-V08B
SARS.CoV_PLpro	SARS-CoV-2	SARS	PLpro	40524-V08E
SARS.CoV_S1.HisTag	SARS-CoV-2	SARS	S1, (His Tag)	40150-V08B1
SARS.CoV_S1.RBD.HisTag	SARS-CoV-2	SARS	S1-RBD, (His Tag)	40150-V08B2
SARS.CoV_S1.RBD.rFcTag	SARS-CoV-2	SARS	S1-RBD, rFc Tag	40150-V31B2
MERS.CoV_NP	MERS	MERS	NP	40068-V08B

MERS.CoV_S1.AA1.725.His.HEK	MERS	MERS	S1, N-(AA1-725, His Tag)_A	40069-V08H
MERS.CoV_S1.RBD.367.606.rFcTag	MERS	MERS	S1-RBD, N-(AA367-606, rFc Tag)	40071-V31B1
MERS.CoV_S1.RBD.383.502.mFcTag	MERS	MERS	S1-RBD, N-(AA383-502, mFc Tag)	40071-V05B
MERS.CoV_S2	MERS	MERS	S2	40070-V08B
DcCoV.HKU23.NP	CommonCoV	HKU23-368F	NP	40458-V08B
hCoV.229E.S1	CommonCoV	229E	S1	UN1-2
hCoV.229E.S1_S2	CommonCoV	229E	S1+S2	UN2-2
hCoV.HKU1.HE	CommonCoV	HKU1	HE	UN1-4-1
hCoV.HKU1.S1_AA1.760	CommonCoV	HKU1	S1, N-(AA1-760)	40021-V08H
hCoV.HKU1.S1_AA13.756	CommonCoV	HKU1	S1, N-(AA13-756)	UN1-3
hCoV.HKU1.S1_S2	CommonCoV	HKU1	S1+S2	UN2-3
hCoV.NL63.S1	CommonCoV	NL63	S1	UN1-1
hCoV.NL63.S1_S2	CommonCoV	NL63	S1+S2	UN2-1
hCoV.OC43.HE	CommonCoV	OC43	HE	UN1-6
hCoV.OC43.S1	CommonCoV	OC43	S1	UN1-5
hCoV.OC43.S1_S2	CommonCoV	OC43	S1+S2	UN2-4
Flu.B_Mal/.HA1	Influenza	B/Malaysia/2506/2004	HA1	11716-V08H1
Flu.B_Mal/.HA1+HA2	Influenza	B/Malaysia/2506/2004	HA1+HA2	11716-V08H
Flu.B_Phu/.HA1	Influenza	B/Phuket/3073/2013	HA1	40498-V08H1
Flu.B_Phu/.HA1+HA2	Influenza	B/Phuket/3073/2013	HA1+HA2	40498-V08B

Flu.H1N1.HA1	Influenza	A/Beijing/22808/2009	HA1	40035-V08H1
Flu.H1N1.HA1+HA2	Influenza	A/Beijing/22808/2009	HA1+HA2	40035-V08H
Flu.H3N2.HA1	Influenza	A/Texas/50/2012	HA1	40354-V08H1
Flu.H3N2.HA1+HA2	Influenza	A/Texas/50/2012	HA1+HA2	40354-V08B
Flu.H5N1.HA1	Influenza	A/Vietnam/1203/2004	HA1	10003-V06H1
Flu.H5N1.HA1+HA2	Influenza	A/Vietnam/1203/2004	HA1+HA2	10003-V06H3
Flu.H7N9.HA1	Influenza	A/Anhui/1/2013	HA1	40103-V08H1
Flu.H7N9.HA1+HA2	Influenza	A/Anhui/1/2013	HA1+HA2	40103-V08H
hAdV3.Fiber	Adenovirus	hAdV-3/45659	Fiber	UN5-9
hAdV3.Penton	Adenovirus	hAdV-3/45659	Penton	UN5-10
hAdV4.Fiber	Adenovirus	hAdV-4/28280	Fiber	UN5-14
hAdV4.Penton	Adenovirus	hAdV-4/28280	Penton	UN5-15
hMPV.A_G.52N.228N	hMPV	PER/CFI0320/2010/A Glycoprotein	hMPV-A_G (52N-228N)	UN6-3
hMPV.B_F.280D.490G	hMPV	PER/CFI0320/2010/A Glycoprotein	hMPV-B_F (280D-490G)	UN6-5
hMPV.B_G.52D.238S	hMPV	PER/CFI0466/2010/B Glycoprotein	hMPV-B_G (52D-238S)	UN6-6
hPIV.1.12O3_F	Parainfluenza	hPIV-1 12O3	F	UN5-1
hPIV.1.12O3_H	Parainfluenza	hPIV-1 12O3	H	UN5-2

hPIV.3.2010_H	Parainfluenza	hPIV-3 USA/10991B/2010	H	UN5-6
hPIV.4.b.2016_H	Parainfluenza	hPIV-4b/10-H2/2016	H	UN5-8
RSV.A.F	RSV	LA2-94/2013	F	UN3-1
RSV.A.G	RSV	LA2-94/2013	G	UN4-1
RSV.B.F	RSV	TH-10526/2014	F	UN3-2
RSV.B.G	RSV	B1	G	13029-V08H
